# Is a Low Dosage of Medical Cannabis Effective for Treating Pain Related to Fibromyalgia? A Pilot Study and Systematic Review

**DOI:** 10.3390/jcm13144088

**Published:** 2024-07-12

**Authors:** Antonio Giardina, Rocco Palmieri, Maria Ponticelli, Carlo Antonelli, Vittorio Carlucci, Monica Colangelo, Nadia Benedetto, Aldo Di Fazio, Luigi Milella

**Affiliations:** 1 Pain Therapy Unit, San Carlo Hospital, Via Potito Petrone, 85100 Potenza, Italy; antonio.giardina@ospedalesancarlo.it (A.G.); rocco.palmieri@ospedalesancarlo.it (R.P.); carlo.antonelli@ospedalesancarlo.it (C.A.); monica.colangelo@ospedalesancarlo.it (M.C.); 2 Department of Science, University of Basilicata, Via dell’Ateneo Lucano 10, 85100 Potenza, Italy; vittorio.carlucci@unibas.it (V.C.); nadia.benedetto@unibas.it (N.B.); 3 Department of Biochemical Pharmacology & Drug Design, Institute of Molecular Biology “Roumen Tsanev”, Bulgarian Academy of Sciences (BAS), Acad. G. Bonchev Str., bl. 21, 1113 Sofia, Bulgaria; 4 Regional Complex Intercompany Institute of Legal Medicine, 85100 Potenza, Italy; aldo.difazio@ospedalesancarlo.it

**Keywords:** medical cannabis, Bedrocan^®^, fibromyalgia, pilot study, systematic review

## Abstract

**Background and Objectives:** Fibromyalgia is a multifaceted and frequently misunderstood chronic pain disease marked by widespread musculoskeletal pain and cognitive/somatic dysfunction. This trial aims to contribute to the existing knowledge on treating fibromyalgia (FM) with medical cannabis (*Cannabis sativa* L.) and explore a safer and more effective cannabis administration method. The goal is to provide evidence-based findings that can guide alternative treatment options for FM patients by assessing a pilot study. **Materials and Methods:** The trial was performed at the pain therapy unit of the San Carlo Hospital (Potenza, Italy) by administrating to 30 FM patients 100 mg/day of Bedrocan^®^ (Bedrocan International, Veendam, The Netherlands) as a decoction. The Numerical Rating Scale (NRS) and SF-12 short-form health questionnaire were used to evaluate pain intensity and the quality of life at the beginning of the study and the 6th-month follow-up. A systematic review of all clinical studies investigating the use of cannabis to reduce FM was also undertaken to place this study in the context of the existing evidence base. **Results:** Pain intensity evaluated with the NRS lowered from a median of 8 [95% CI 7.66–8.54] at a baseline to a median of 4 (95% CI 3.28–4.79) after 6 months of follow-up (*p*-value < 0.001; *t*-test). Similarly, significant physical and mental state improvement, evaluated with the SF-12 questionnaire, was found in 96.67% and 82.33% of patients, respectively (95% CI 44.11–51.13 for the physical state, and 53.48–58.69 for mental state assessed after the 6th-month follow-up; *p*-value < 0.001; *t*-test). The systematic analysis of the literature identified 10 clinical trials concerning the treatment of fibromyalgia with cannabis. **Conclusions:** Considering results from the present pilot study and systematic review, it is possible to assume that medical cannabis may be considered an alternative therapy for FM patients who do not respond to conventional pharmacological therapy.

## 1. Introduction

**Figure 1:**
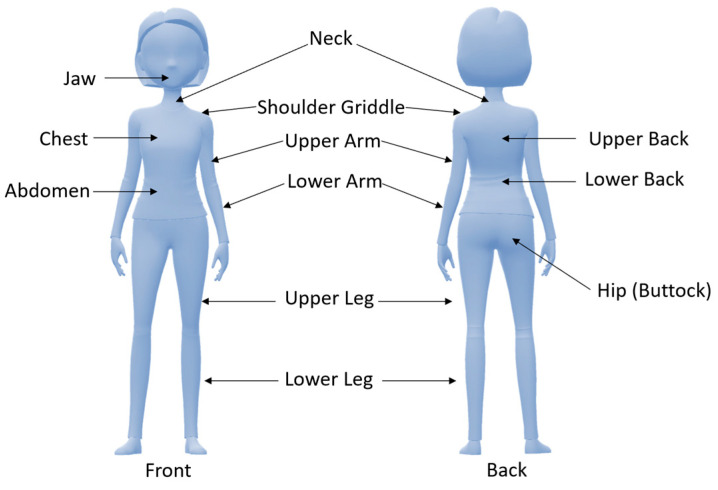
Tender points used for the diagnosis of fibromyalgia with the widespread pain index (WPI).

Fibromyalgia (FM) is a term introduced in the early 1970s denoting a multifaceted and challenging clinical condition that transcends conventional medical classifications. FM definition has been revised significantly over the years, reflecting the evolving understanding of the condition. Initially considered mainly as a rheumatic disorder, it is now acknowledged as a pain processing disorder and sensitization of the central nervous system [1]. FM is, indeed, one of the most common causes of persistent chronic widespread pain (CWP). However, although pain is its main feature, it is represented by a complex polysymptomatology comprising fatigue, sleep disturbances, generalized hyperalgesia, stiffness, palpation-specific tender points, and cognitive and somatic dysfunction [2]. Pain might be initially generalized or localized in a specific body region, such as the neck or lower back. Fatigue is chronic in FM patients and is frequently referred to as moderate to severe, while cognitive problems are informally reported as part of the “*fibro fog*” and consist of compromised memory, attention, and concentration [3]. Due to FM’s multifaceted and complex nature, it poses a significant diagnostic challenge for doctors. For this reason, an FM diagnosis takes about two years, and patients usually have to see several medical specialists during this time [4]. Failure to diagnose FM in the past was exacerbated by the medical community’s skepticism, considering it as a psychosomatic disorder. This skepticism has hindered the recognition and understanding of FM due to the lack of objective diagnostic criteria and markers. However, as studies progressed, an expanding body of evidence illuminated the intricate interplay of psychological, biological, and social factors influencing the syndrome outbreak [1]. Nowadays, several diagnostic criteria have been developed, but those approved by the American College of Rheumatology (ACR) and first introduced in 1990 are normally used. Following the ACR, for the FM diagnosis, pain must be triggered by exerting pressure (tender points) up to 4 kg/cm^2^ in at least 11 of the 18 specified body points, and patients must have a multisite or widespread pain lasting more than 3 months [4]. In this context, widespread pain was labeled as right- and left-side pain, axial pain, and lower- and upper-segment pain [5]. However, the ACR classification criteria did not consider symptoms recently related to FM, such as cognitive problems and somatic symptoms. For this reason, in 2010, two variables best defined FM and its symptom aspect were identified: the widespread pain index (WPI) and the symptom severity (SS) scale. The WPI is strongly related to nineteen tender points (Figure 1), while the SS scale evaluates fatigue grade, unrefreshed sleep, and physician-rated cognitive symptoms (Table 1).

These two criteria were combined to define a new FM case definition: WPI ≥ 7 and SS ≥ 7 or WPI 3–6 and SS ≥ 9 [5].

For FM, a multidisciplinary intervention is advocated since pharmacological treatments are generally combined with approaches involving psychotherapy, patient education, physiotherapy, and pharmacological treatments [2,6]. The commonly prescribed drugs are antidepressants, anticonvulsants, muscle relaxants, analgesics, hypnotics, and antipsychotics [6,7]. Table 2 lists the main drugs used to treat FM [8,9].

However, many of these drugs offer few benefits and are frequently associated with side effects that may reduce patient compliance [10]. In the last year, particular attention was paid to the use of natural products in clinical practice [11,12,13]; specifically, cannabinoid-containing products were investigated in clinical settings due to their anti-inflammatory and immunomodulatory activity, making them useful for managing pain-associated symptoms [14]. The current literature has indeed demonstrated that medicinal cannabis (*Cannabis sativa* L.) can be associated with an improved quality of life for patients suffering from chronic pain [15]. Two different types of cannabinoid-containing products exist on the market: isolated compounds [delta-9-tetrahydrocannabinol (THC) and cannabidiol (CBD)] and plant-based (cannabis) products. In the present 6-month pilot study, the effect of a plant-based formulation (cannabis: Bedrocan^®^, 22% THC, <1% CBD) has been studied on a sample of 30 adult patients with FM resistant to opioid therapy. The choice of testing a plant-based formulation, and not an isolated compound, was made considering that cannabis, besides THC and CBD, contains other cannabinoid and non-cannabinoid molecules like terpenes that can synergically contribute to reducing pain [16,17]. In fact, for the so-called “entourage effect”, terpenes interact with cannabinoids, improving their pharmacodynamic effects by increasing cannabinoids’ charge binding to their receptors and pulmonary uptake [18]. Further, terpenes have their own effects, like analgesic, anti-inflammatory, and antidepressant effects [19]. Therefore, this study aims to evaluate the effectiveness of cannabis (Bedrocan^®^) therapy in fibromyalgia patients by analyzing their quality of life, as measured by the SF-12 short-form health questionnaire and by measuring pain intensity with the Numerical Pain Rating Scale (NRS). In addition, to place this study in the context of the existing evidence base, a systematic review of all clinical studies investigating the use of cannabis to reduce FM was also undertaken.

## 2. Materials and Methods

### 2.1. Study Design, Setting, and Outcomes

The current clinical trial is a pilot study (ClinicalTrials.gov Identifier: NCT05939466) investigating the analgesic effect of medical cannabis (MC) in adult Italian patients with a diagnosis of FM and who reported pain resistance to pharmacological treatments (e.g., Patrol, Busette, Arcoxia, Dulex, Depalgos, etc.).

Eligible subjects were those visiting the pain therapy unit of the San Carlo Hospital (Potenza, Italy) and fitted with the following inclusion criteria: informed written consent; age >18 years old; diagnosis of FM confirmed by a rheumatologist; persistent pain symptoms for at least three months without complaints that may otherwise explain the pain condition; persistent pain syndrome on conventional therapy with opioids or non-steroidal anti-inflammatory drugs; not having taken MC in the previous year since the start of the study; stopping drug therapy during the trial with cannabis (Bedrocan^®^).

Subjects were excluded from the trial if they agreed with the following criteria: specific contraindications to cannabinoid use; pain syndrome not associated with FM; major comorbidities like renal impairment, severe liver disease, chronic hepatitis C, history of alcohol or drug addiction; the presence of cognitive deficits that could impair understanding of the study, completion of questionnaires, or adherence to therapy; pregnant or planning pregnancy women and breastfeeding women.

**Figure 2:**
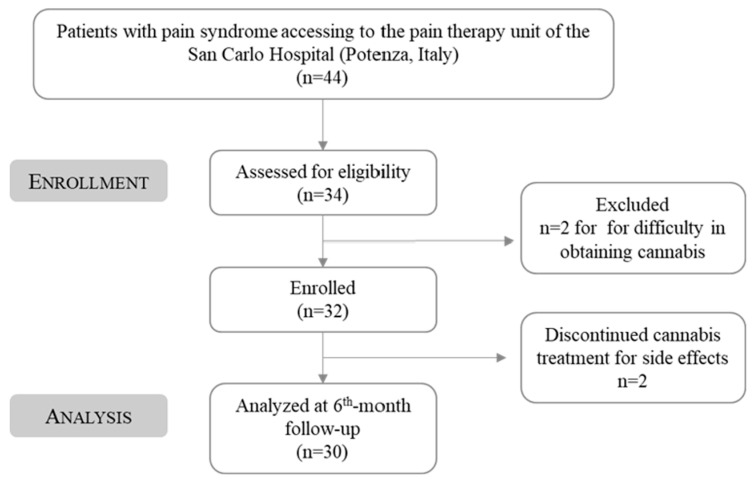
Study flow-chart diagram.

This study was approved by the San Carlo Hospital ethics committee (n. of CEUR general register 37/2023, n. protocol of the Potenza’s AOR San Carlo referring to application for approval 20230021536, protocol date 11 May 2023) and was conducted in the pain therapy unit of the San Carlo Hospital (Potenza, Italy) from March 2021 to September 2021. During this period, 44 subjects who suffered from pain syndrome came to the pain therapy unit and were examined by a specialist. The presence of FM syndrome was validated by using the WPI and the SS scale (WPI ≥ 7 e SS ≥ 5 o WPI 3–6 e SS >9). Eligible subjects who provided informed written consent for starting MC treatment were given the prescription for Bedrocan^®^ once a month for a total of 30 charts per month for 6 months. Specifically, 34 patients were recruited at the beginning of the study. However, 2 subjects did not start therapy in time to be included in the present study and were excluded, while 2 other patients dropped out of therapy due to side effects. Thus, the total number of analyzed FM subjects was 30 (Figure 2).

Each subject was assigned a numerical identification code to ensure the patient’s anonymity.

**Figure 3:**
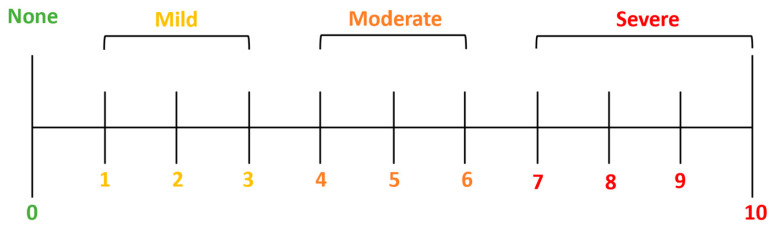
Numerical Pain Rating Scale (NRS).

The study’s primary outcomes include the evaluation of the Numerical Pain Rating Scale (NRS), a valuable tool in clinical practice for assessing and monitoring pain levels, guiding treatment decisions, and evaluating the effectiveness of pain management interventions (Figure 3).

The study’s secondary outcomes include the evaluation of the short-form health survey (SF-12), a valuable tool for assessing health status and evaluating the impact of interventions on physical and mental well-being in clinical and research settings. The SF-12 consists of 12 questions that cover eight health domains: physical functioning, role limitations due to physical health, bodily pain, general health perceptions, vitality (energy/fatigue), social functioning, role limitations due to emotional problems, and mental health (psychological distress and well-being). Responses to the SF-12 are scored using a standardized method to derive two summary scores: physical component summary (PCS), reflecting the overall physical health status, and mental component summary (MCS), indicating the overall mental health status.

### 2.2. Treatment Regimen

Bedrocan^®^-type cannabis (22% THC, <1% CBD) was used because, compared to other formulations such as FM2 and Bediol (Bedrocan International), it ensured a more constant supply in Lucanian pharmacies, reducing the risk of therapy interruption due to the lack of availability of the drug. The MC was administered with herbal tea or decoction, as described by the Ministry of Health, through the Ministerial Decree of 9 November 2015. In the case of the present study, the Dutch protocol was used, which, in addition to the Italian protocol, provides for adding milk to form a lipophilic emulsion capable of increasing the extraction of the cannabinoids.

The nurses instructed the patients on how to prepare the decoction, as described below. Place the inflorescences and water in a container at the following ratio: 100 mL cold water for every 100 mg of cannabis. Heat to a boil and simmer for 15 min, stirring regularly. Allow the decoction to cool for about 15 min, then stir and strain by pressing the remaining residue on the filter with a spoon to recover more liquid and enrich the final solution. If the decoction is not consumed at the time of preparation, it can be stored in a closed container in the refrigerator for up to 24 h. When cannabis is taken orally by ingesting it, it takes 30–90 min before the effects are felt, maximum after about two to three hours and minimum after about six hours.

The therapy with Bedrocan^®^ was started with 100 mg/day (1 chart) and was increased to 2 charts (200 mg/day) at the first-month follow-up in non-responsive patients.

### 2.3. Statistical Analysis

For the analysis of the data concerning the SF-12 questionnaire, the free source code made available by the Istituto di Ricerche Farmacologiche Mario Negri of Milan in Annex 4 of the manual *Questionarioe Sullo Stato Di Salute SF-12 Italian version* [20]. All clinical information collected during the clinical trial was stored and analyzed using Microsoft Excel (Microsoft Office Professional Plus 2021) and Minitab^®^ (version 19). The final analysis was performed per protocol (PP) since only data from subjects who strictly adhered to the study protocol were analyzed. All data were expressed as median, interquartile range calculated at 25° percentile, mean ± standard deviation (SD), and 95% interval confidence (95% IC). Results with a *p*-value < 0.001 were considered significant.

### 2.4. Systematic Review

Following the Preferred Reporting Items for Systematic Reviews and Meta-Analyses (PRISMA) guidelines, a systematic literature search was performed in March 2023, including all clinical studies published until April 2023. Scopus (https://www.scopus.com/standard/marketing.uri) and PubMed (https://pubmed.ncbi.nlm.nih.gov/) were used as research databases. Only full texts were included, and those not available were not requested, as keywords were utilized: “cannabis” and “fibromyalgia”; “cannabis” and “fibromyalgia” and “Clinical trial”; “cannabis” and “fibromyalgia” and “Observational study”; “cannabis” and “fibromyalgia” and “Randomized study”.

The systematic research was performed by including only clinical investigation involving the use of cannabis for Fibromyalgia syndrome treatment. Only articles written in English and containing the keyword in the abstract or title were included, while review articles, meta-analyses, letters, editorials, abstracts, and manuscripts with a not available text were not used for writing the systematic review. Two independent investigators (Rocco Palmieri and Maria Ponticelli) selected documents based on the title and abstract adequacy and subsequently analyzed all full texts. In case of disagreement, other independent reviewers were involved (Luigi Milella and Antonio Giardina). All articles selected were closely reviewed for the inclusion or exclusion of manuscripts that did not comply with the described criteria.

#### Methodological Quality Assessment

Articles quality assessment was performed considering the Cochrane Methods Bias (https://www.riskofbias.info/welcome/home) for clinical studies. Specifically, the risk of bias evaluation was performed considering the absence or presence of the information reported in the Cochrane Handbook for Systematic Reviews of Interventions for randomized and non-randomized studies [21,22]. The robvis tool, including ROB2 for randomized clinical studies [23] and ROBINS-1 for non-randomized clinical study [24], was used to organize the data.

## 3. Results

### 3.1. Patients Characteristics

Forty-four patients (34 women and 10 men) with a median age of 51.5 years were visited for pain conditions in the pain therapy unit of the San Carlo Hospital (Potenza, Italy). Among these 44 subjects, 34 (28 women and 6 men) were considered eligible for study participation since a correlation between pain and FM was diagnosed. Two subjects, due to supply problems, did not start therapy in time to be included in the present study and were excluded, while two other patients dropped out of therapy due to side effects (both due to psychomotor agitation) at the first administration and were excluded from the study. Psychomotor agitation appeared with repetitive, involuntary movements, a condition that may be related to the active ingredient tested. It is indeed seen that while cannabis is commonly associated with relaxation and sedation, it can also induce psychomotor agitation in some individuals, especially in susceptible ones [25].

The total number of recruited and analyzed FM subjects was 30 (24 women and 6 men) with a median age of 50.5 years (range 32–82 years): 12.5% of subjects were <40 years old, 25% were 41–50 years old, 25% were 51–69 years old, and 12.5% were >70 years old (Table 3).

Comorbidities and pain syndromes associated with FM were evaluated for all considered subjects. The main comorbidities were hypertension (20%) and hypothyroidism (10%), while the most frequent pain syndromes associated with FM were polydistractual pain (40%) and migraine (6.67%) (Table 4).

The most assumed therapies included for reducing FM symptoms were opiates (60%), non-steroidal anti-inflammatory drugs (23.33%), and antidepressants (23.33%). Before starting the current trial, all patients stopped to assume the drug therapy they were following.

### 3.2. Numerical Pain Rating Scale (NRS)

The NRS is commonly used in a clinical setting to evaluate patients’ pain intensity through a pain scale from 0 to 10, where 0 indicates “no pain” while 10 is “the worst imaginable pain”. The present study evaluated the pain intensity at baseline and the sixth month of follow-up (Table 5).

**Figure 4:**
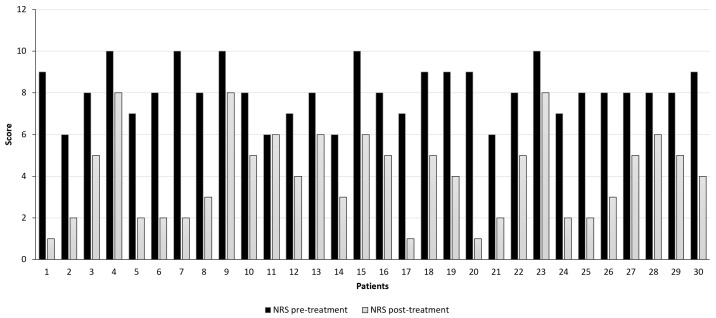
Pain intensity assessment was evaluated with the Numerical Pain Rating Scale (NRS) before (black bars) and after 6 months (grey bars) of therapy with Bedrocan^®^ decoction. (*p*-value < 0.001; *t*-test).

Prior to the treatment with MC, 24 patients (80%) reported an NRS score comprised between 8 and 10. As reported in Figure 4, after 6 months, only 3 patients (10%) reported a mild pain improvement (NRS score from 10 to 8 at 6th-month follow-up), while 1 subject (3.33%) did not show pain amelioration (NRS score of 6 at baseline and after 6th-month follow-up).

Overall, pain intensity lowered from a median of 8 [95% confidence interval (CI) 7.66–8.54] at a baseline to a median of 4 (95% CI 3.28–4.79) after 6 months of follow-up (*p*-value < 0.001; *t*-test). Hence, a total NRS score reduction of 50% was evidenced after 6 months of treatment with Bedrocan^®^.

### 3.3. Short-Form Health Survey (SF-12)

The SF-12 is a questionnaire designed to investigate the perception of individuals’ psychophysical conditions. The synthesis of the scores makes it possible to construct two health status indices, one concerning the physical state (physical component summary, PCS-12) and the other the psychological state (mental component summary, MCS-12). Synthetic indices values may vary from 10.5 to 69.7 for the PCS and 7.4 to 72.1 for the MCS index, where psychophysical health conditions improve as they increase. PCS levels, approximately below 20 points, correspond to a condition of “substantial limitations in self-care and physical, social and personal activity; significant physical pain; frequent fatigue; poor health”. Similarly, a low psychological health index shows “frequent psychological distress; significant social and personal impairment due to emotional problems; poor health” [26].

**Figure 5:**
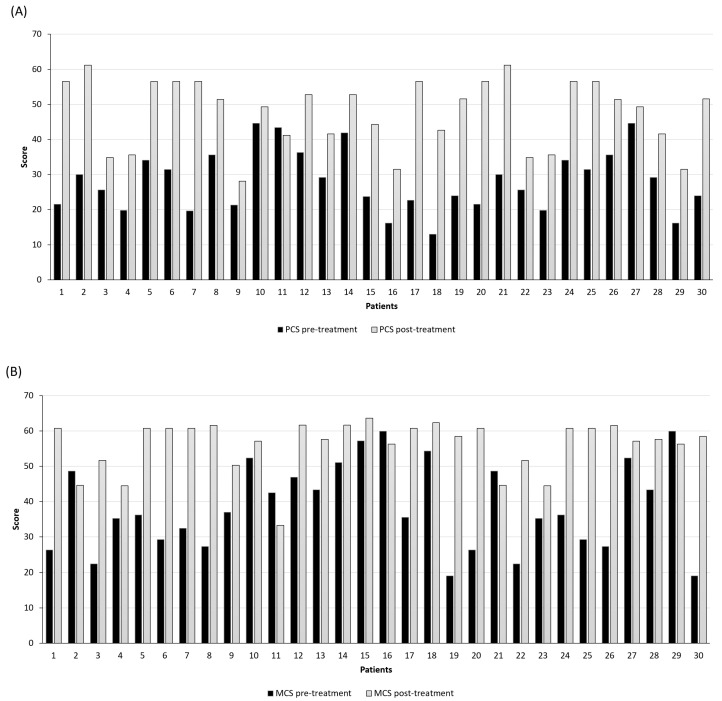
Evaluation of the quality of life with the short-form health survey (SF-12): (**A**) physical component summary, PCS-12; (**B**) mental component, MCS-12. Black bars represent the score before starting the treatment, while grey bars indicate the score at the 6th month of follow-up (*p*-value < 0.001; *t*-test).

Regarding the physical state evaluation before the treatment with Bedrocan^®^, 20.00% of patients reported a PCS score lower than 20, indicative of a scarce quality of life (Table 3; Figure 5).

However, after treatment, a significant quality of life improvement was found in 96.67% of patients with a median of 27.40 at baseline and 51.46 after 6th-month follow-up (95% CI 25.09–31.33 and 44.11–51.13, respectively; *p*-value < 0.001; *t*-test). Contrarily, 1 subject (3.33%) assessed only a slight reduction in the PCS score (from 43.44 at baseline to 41.21 after 6 months).

Regarding the physical state, a significant mental state amelioration was seen for 83.33% of patients, with a median of 36.22 at baseline and 58.46 after the 6th month of follow-up (95% CI 34.12–43.00 and 53.48–58.69, respectively; *p*-value < 0.001; *t*-test).

### 3.4. Collateral Effects

During the treatment with cannabis, 2 patients out of 32 first included subjects (6.25%) dropped out of the study due to side effects related to psychomotor agitation. The remaining 30 patients (93.75%) who completed the study up to 6 months experienced no side effects.

### 3.5. Systematic Review

**Figure 6:**
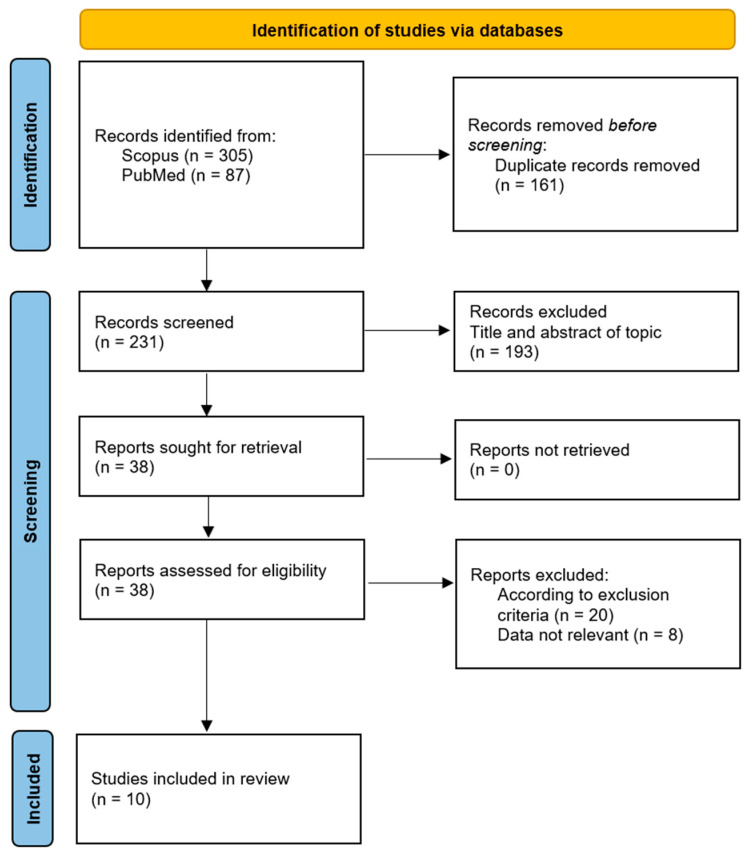
Systematic review literature research based on Preferred Reporting Items for Systematic Reviews and Meta-Analyses (PRISMA) statements [27,28].

To insert this study in the context of the existing clinical trials, a systematic review of the literature was performed. Specifically, the systematic review was performed by following the PRISMA statement recommendation [27], including studies published between 2000 and 2023 using Scopus and Pubmed as databases. The initial manuscripts selection provided 392 studies, of which 305 were found on Scopus and 87 on PubMed. Among the 392 studies, 161 are duplicates, resulting in 231 studies out of those 213 not fitting with the considered inclusion criteria and 8 not containing information congruent with the study topic. Thus, the final reference list includes 10 clinical studies (Figure 6), of which 8 observational studies and 2 randomized clinical trials have been published since 2000 regarding the use of cannabis in the treatment of FM.

Overall, as reported in the present study, a significant reduction in pain and an amelioration of quality of life were recorded. Table 6 summarizes the data from the included clinical trials.

#### Risk of Bias Assessment

**Figure 7:**
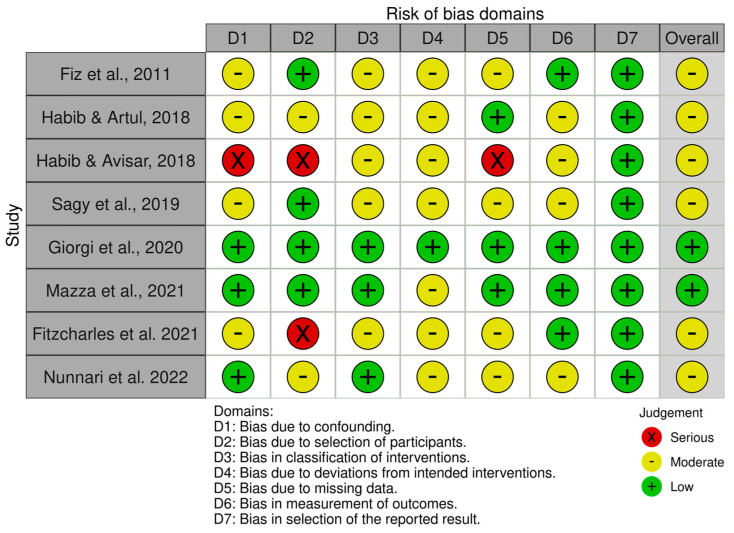
Risk of Bias analysis for non-randomized studies made using the ROBINS-1 tool (https://www.riskofbias.info/welcome/home) [29,30,31,32,34,36,37,38].

**Figure 8:**
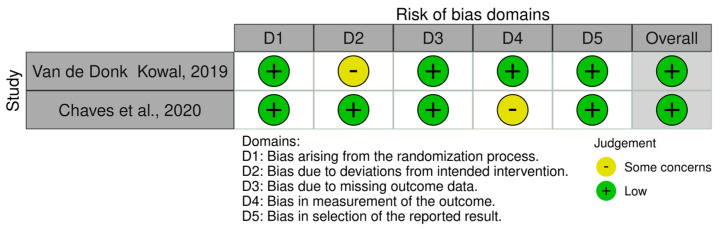
Risk of Bias analysis for randomized studies made using the ROB2 tool (https://www.riskofbias.info/welcome/rob-2-0-tool) [33,35].

Detailed results of the risk of bias analysis are reported in Figure 7 and Figure 8. Of the eight non-randomized clinical trials (Figure 7), six have an overall medium risk of bias predominantly due to bias in the classification of intervention (D3) and deviation from the intended intervention (D4). On the contrary, the two randomized clinical trials have a low risk of bias (Figure 8).

## 4. Discussion

In this study, the efficacy of MC in FM patients was evaluated, obtaining results that suggest a possible implication of MC as an alternative treatment for patients with FM not responsive to conventional pharmacological therapy. As in other studies [29,30,31,32,36], in the current pilot study, most of the patients were women since they represent 89.3% of the patients. Considering that, as previously stated, the male-to-female ratio with FM syndrome is 1:3 [39], the current investigation confirmed that FM patients with clinical pain are women. Noteworthy is the NRS at baseline since patients with a pain score comprised between 8 and 10, representing the maximum level of pain, were 24 (80% of subjects). This basal pain score is higher than that registered in other clinical trials investigating the effect of a synthetic derivative of Δ-9-THC, nabilone [40,41], while it is in line with that registered by Mazza et al. and Sagy et al. [32,36]. As in the last clinical study [32], the current one revealed a significant reduction in NRS from a median of 8 (95% CI 7.66–8.54) at a baseline to a median of 4 (95% CI 3.28–4.79) after 6 months (*p*-value < 0.001; *t*-test). Hence, there was a reduction in pain score in the 6th-month follow-up of 4 points, comparable to the pain decrease that was assessed by Mazza et al. in the 12th month (4.3 points) [36]. An improvement in pain symptoms was also assessed in two observational studies [30,34] and two randomized, double-blind, placebo-controlled clinical trials [33,35]. Even if the data obtained in these investigations are not comparable with those of the current one since another evaluation parameter was used, the Fibromyalgia Impact Questionnaire (FIQ) and the pressure pain test. Finally, a reduction in pain evaluated with the VAS score was detected in an observational cross-sectional survey investigating the short-term effect of cannabis inhalation, while only a low analgesic effect was detected [29]. In the complex, these results may indicate a potential application of cannabis in reducing pain. In the current pilot study, the improvement of pain symptoms was corroborated by the data from the SF-12 consisting of 12 items (taken from the 36 items of the original SF-36 questionnaire) that produce two measures relating to two different aspects of health: physical health (PCS-12) and mental health (MCS-12). In the current trial, the PCS-12 was significantly improved from the baseline to the 6th month for 96.67% of FM subjects [mean (M) = 28.21 ± 8.72 to 47.62 ± 9.81 for baseline and 6th-month evaluation, respectively, *p*-value < 0.001; *t*-test]. In the same way, the MCS-12 increase significantly for 83.33% of FM patients (M = 38.56 ± 12.41 to 56.09 ± 7.28 for baseline and 6th-month evaluation, respectively, *p*-value < 0.001; *t*-test). These results are indicative of an improvement in the quality of life and are in line with those of previous clinical trials since an increase in mental score was also seen when it was evaluated with the SF-36 [29]. SF-36 is the SF-12 extended version and has been demonstrated to be complementary to the SF-12, confirming the comparable nature of results obtained from the SF-36 and SF-12 questionnaire [42]. Enhanced mental health was further evidenced in another survey, where 85% and 62% of FM patients ameliorated depressive and anxious states after cannabis assumption [31]. Contrarily, no differences in mental mood were observed when two synthetic Δ-9-THC derivatives, nabilone and amitriptyline, were administered [43]. Improved quality of life was also seen in two more observational studies when it was evaluated with the Likert scale regarding appetite, sleep quality, and sexual activity [15,32]. Thus, based on these results, it is possible to assert the potential therapeutic effect of cannabis for the treatment of FM. This assumption was further supported by the demonstrated safety of MC taken as decoction since only 6.25% of subject reported psychomotor agitation, while no other side effects were reported.

One strength of the current Pilot Study was the evaluation of the cannabis effect in a controlled hospital setting by using only a type of administration mood and not a combination of administration mood (e.g., decoction and oil or smoke) to reduce the risk of bias. Furthermore, it was demonstrated that decoction is patients’ preferred cannabis mode of assumption since it is easy to prepare and has a good safety profile, thus avoiding accidental overdoses of THC and the consequent psychotropic effect [44]. Another strength was the low dosage of 100 mg (1 sachet) per day, which was increased to 200 mg (2 sachets) per day for only two patients (7.14%) who were not responsive to the low dosage. This administered cannabis amount, corresponding approximately to 3.0 g per month, was definitively lower than that used in other investigations (about 31.4 ± 16.3 g per month) [30,31,32,36]. Finally, in the present clinical study, before starting MC therapy, all patients stopped the assumption of conventional drugs, while in all other studies, the pharmacological therapy was continued during the evaluation of MC efficacy [30,32,34,35]. This may suggest that MC can represent a suitable and efficacious alternative to conventional drugs. However, due to the small number of FM patients investigated, the risk of bias is high, and the results obtained should be interpreted carefully. It is also important to take into consideration the limitations of this study since the use of self-administered questionnaires may represent a source of bias. Furthermore, the absence of a control group in the present investigation can represent an additional limitation. Hence, further randomized placebo control clinical trials with a higher number of participants should be needed to better assess the role of MC, administered as a decoction, in FM symptom reduction.

## 5. Conclusions

FM is one of the most common causes of persistent chronic and widespread pain. However, although pain is its main feature, it is represented by a complex polysymptomatology comprising fatigue, sleep disturbances, generalized hyperalgesia, stiffness, palpation-specific tender points, and cognitive and somatic dysfunction. The current pilot study evidenced a positive effect of a low dosage of MC (Bedrocan^®^; 100 mg/day) in treating FM symptomatology. Likewise, data from the literature demonstrated that cannabis administration could be associated with an improved quality of life for patients suffering from chronic pain. Hence, it is possible to conclude that cannabinoids may represent an effective alternative to conventional pharmacological therapy for reducing pain and mind disorders in FM subjects. Further investigations like randomized, placebo-controlled clinical trials are needed to corroborate these findings.

## Figures and Tables

**Table 1:** Symptom severity (SS) scale.

	No Problem	Mild	Moderate	Severe
**Difficulty thinking or remembering**	0	1	2	3
**Awakening tired (not rested)**	0	1	2	3
**Fatigue**	0	1	2	3

**Table 2:** FM pharmacological treatment.

	Class
**FDA approved drug**	
Duloxetine	Serotonin–Norepinephrine Reuptake Inhibitor (SNRI)
Milnacipram	Serotonin–Norepinephrine Reuptake Inhibitor (SNRI)
Pregabalin	Anticonvulsant/Neuropathic Pain Agent
**Commonly used drug**	
Amitriptyline	Tricyclic Antidepressant (TCA)
Cyclobenzaprine	Muscle Relaxant
Gabapentin	Anticonvulsant/Neuropathic Pain Agent
Naltrexone	Opioid Antagonist
Acetaminophen	Analgesic

**Table 3:** Patients characteristics.

Parameters	n = 30			
**Gender**	**Male**	20%		
	**Female**	80%		
	**Mediana**	**IQR**	**Mean ± SD**	**95% CI**
**Age**	50.50	45.75	53.17 ± 13.07	48.49–57.84
**Weight**	70.00	67.75	73.40 ± 10.28	69.72–77.08
**BMI (kg/m^2^)**	26.14	24.77	26.80 ± 3.89	25.41–28.19
**WPI**	8.00	6.00	7.53 ± 2.16	6.76–8.31
**SS**	7.00	5.00	7.10 ± 1.88	6.43–7.77

Abbreviations: IQR, interquartile calculated at the 25th percentile; ± SD, plus/minus standard deviation; 95% CI, 95% interval of confidence; BMI, body mass index (kg/m^2^); WPI, widespread pain index; SS, SyS.

**Table 4:** Comorbidities and pain syndrome associated with FM.

Comorbidities			Pain Syndrome Associated with FM		
	Patients	%		Patients	%
**Hypertension**	6	20	**Polydistrict pain**	12	40
**Hypothyroidism**	3	10	**Migraine**	2	6.67
**Hereditary thrombophilia**	1	3.33	**Arthrosis**	1	3.33
**Convers syndrome**	1	3.33	**Rhizarthrosis**	1	3.33
**Hypercholesterol**	1	3.33	**Insomnia**	1	3.33
**Migraine**	1	3.33	**Trigeminal pain**	1	3.33
**Dyspeptic syndrome**	1	3.33	**Headache**	1	3.33
**Obesity**	1	3.33	**FBSS**	1	3.33
**Heart disease**	1	3.33			
**Scleroderma**	1	3.33			
**Raynaud’s disease**	1	3.33			

Abbreviations: FM, fibromyalgia; FBSS, Failed Back Surgery Syndrome.

**Table 5:** Effect of medical cannabis (MC), Bedrocan^®^, on pain assessment and quality of life before treatment (Prior MC) and at 6th month of follow-up (After MC).

	NRS			SF-12					
				PCS-12			MCS-12		
	Prior MC	After MC	*p*-Value	Prior MC	After MC	*p*-Value	Prior MC	After MC	*p*-Value
**Median**	8.00	4.00	<0.001	27.40	51.46	<0.001	36.22	58.46	<0.001
**IQR**	7.00	2.00		21.49	39.80		27.29	51.65	
**Mean**	8.10	4.03		28.21	47.62		38.56	56.09	
**±SD**	1.24	2.11		8.72	9.81		12.41	7.28	
**95% CI**	7.66–8.54	3.28–4.79		25.09–31.33	44.11–51.13		34.12–43.00	53.48–58.69	

Abbreviations: NRS, Numerical Pain Rating Scale; SF-12, short-form health survey; PCS-12, physical component summary; MCS-12, mental component summary; IQR, interquartile calculated at the 25th percentile; ±SD, plus/minus standard deviation; 95% CI, 95% interval of confidence.

**Table 6:** Clinical trials included in the systematic review.

Author and Reference	Type of Study	Duration of the Study	Number of Investigated Subjects	Type of Cannabis Administered	Route of Administration	Evaluation Modality	Outcomes
Fiz et al., 2011 [29]	Observational (cross-sectional survey)	1 day Data were taken at baseline and after 2 h of cannabis administration	28 93% women	Not tirtrated cannabis	Smoked (54%), oral (46%) combined (43%).	VAS FIQ PSQI SF-36	↓VAS score ↓pain and stiffness ↑relaxation ↑feeling well being ↑SF-36 mental component ~SF-36 phisical component ~FIQ ~PSQI
Habib and Artul, 2018 [30]	Observational (Retrospective)	2 months	26 73% women	Not tirtrated cannabis 26 ± 8.2 g/month	Smoked (58%) Vaporized (23%) Vaporized + Smoked (14%) Oral oil drop + Smoked (8%)	Revised FIQ	Improvement of all parameters evaluated with the revised FIQ
Habib and Avisar, 2018 [31]	Observational (Retrospective)	Not specified	383 85% women	Not titrated cannabis 31.4 ± 16.3 g/month	Smoked with tobacco 63% Smoked pure cannabis 17% Sublingual oil drop 5% Vaporized 15%	Evaluation of daily activity, pain relief, slip quality, anxiety, and depression	Improvement of all evaluated parameters
Sagy et al., 2019 [32]	Observational	6 month	211	THC- or CBD-rich strains 670 mg/day of cannabis at the beginning and 700 to 1000 mg at six months Median THC at six months 140 mg/day Madian CBD at six months 39 mg/day	Oil drops Inflorescence Capsules Cigarettes (the specific percentage of patients that used one or the other mode of administration was not specified)	NRS 5 and 8 point Linkert scale	↑Sleep problems ↓Depression ↑Quality life
Van de Donk Kowal, 2019 [33]	Randomised Placebo-Controlled 4-Way Crossover Trial	Not specified (patients received 1 of the 4 available type of cannabis with a minimum 2-week interval between visits)	25 female patients	Bedrocan^®^: 22.4 mg THC, <1 mg CBD Bediol^®^: 13.4 mg THC, 17.8 mg CBD. Bedrolite^®^ (Bedrocan International): <1 mg THC, 18.4 mg CBD. Placebo One inhalation.	Vaporization	Pressure pain test Electrical pain test Bowdle questionnaire Bond and Lader questionnaire	↑pressure pain threshold for cannabis variety containing THC ↓pain score of 30% for Bediol compared to placebo
Giorgi et al., 2020 [34]	Observational (Perspective)	6 month	102 91% women	Bedrocan^®^: 22% THC, <1% CBD Bediol^®^: 6.3% THC, 8% CBD.	Oil oral drops	FAS Revised FIQ PSQI SAPS ZSR-D ZSR-A FACIT	↑PSQI in 44% of subjects ↓FIQ score in 33% of patients Moderate improvement in ZSR-D and ZRS-A in 50% of subjects
Chaves et al., 2020 [35]	Randomised Double Bling Placebo-Controlled Clinical trial	8 weeks	17 female patients	White Widow variety - 48:1 THC:CBD Placebo 1 drop/day	Sublingual oil drop	FIQ	↓FIQ score in cannabis group compared to placebo
Mazza et al., 2021 [36]	Observational (Retrospective)	12 months	38 95% women	FM2 5–8% THC, 7.5–12% CBD Bediol 6% THC, 8% CBD FM1 13–20% THC, <1% CBD Bedrocan 22% THC, < 1% CBD Pedanios 17–26% THC, <1% CBD THC-dominant variety minimum 50 mg/day; maximum 600 mg/day Hybrid cannabis variety minimum 100 mg/day, maximum 600 mg/day	Oral decoction Sublingual oil drop Vaporized	ODI HADS SyS	1 month follow up ↓NRS, ODI, WPI and severity score 3 months follow up ↓NRS, ODI, WPI 12 months follow up ↓NRS, ODI, and severity score
Fitzcharles et al., 2021 [37]	Observational	2 months	117 91.5% women	Prescribed and not prescribed cannabis For inhaled cannabis 0.5 to 2 g per day, mostly	Smoked Vaporized Oil	VAS PtGA PGA	Pain syndrome relief
Nunnari et al., 2022 [38]	Observational (Retrospective)	The median duration of cannabis consumption is 12 months	56 73.2% women	Bedrocan 78.5% of patients Bediol 17.9% of patients FM2 3.6% of patients	Cannabis oil	Effect of cannabis administration on pain medication discontinuation	Reduction in opioid users

Abbreviations: NRS, Numerical Pain Rating Scale; SF-36, short-form health survey; ODI, Oswestry Disability Index; WPI, widespread pain index; SyS, severity score; VAS, visual analog scales; FIQ, Fibromyalgia Impact Questionnaire; FAS, Fibromyalgia Assessment Scale; PSQI, Pittsburgh Sleep Quality Index; HADS, Hospital Anxiety and Depression Scale; SAPS, Self-Assessment Pain Scale; ZSR-D, Zung Self-Rating Depression Scale; FACIT, Functional Assessment of Chronic Illness Therapy; ↓ Reduction; ↑ Increase.

## Data Availability

The original contributions presented in the study are included in the article, further inquiries can be directed to the corresponding authors.
